# Renal effects of the serine protease inhibitor aprotinin in healthy conscious mice

**DOI:** 10.1038/s41401-021-00628-1

**Published:** 2021-03-23

**Authors:** Stefan Wörner, Bernhard N. Bohnert, Matthias Wörn, Mengyun Xiao, Andrea Janessa, Andreas L. Birkenfeld, Kerstin Amann, Christoph Daniel, Ferruh Artunc

**Affiliations:** 1 Division of Endocrinology, Diabetology and Nephrology, Department of Internal Medicine, University Hospital Tübingen, Tübingen, Germany; 2 Institute of Diabetes Research and Metabolic Diseases (IDM) of the Helmholtz Center Munich at the University Tübingen, Tübingen, Germany; 3 German Center for Diabetes Research (DZD) at the University Tübingen, Tübingen, Germany; 4 Department of Nephropathology, Friedrich-Alexander University (FAU) Erlangen-Nürnberg (FAU), Erlangen, Germany

**Keywords:** aprotinin, serine proteases, renal dysfunction, epithelial sodium channel, sodium

## Abstract

Treatment with aprotinin, a broad-spectrum serine protease inhibitor with a molecular weight of 6512 Da, was associated with acute kidney injury, which was one of the reasons for withdrawal from the market in 2007. Inhibition of renal serine proteases regulating the epithelial sodium channel ENaC could be a possible mechanism. Herein, we studied the effect of aprotinin in wild-type 129S1/SvImJ mice on sodium handling, tubular function, and integrity under a control and low-salt diet. Mice were studied in metabolic cages, and aprotinin was delivered by subcutaneously implanted sustained release pellets (2 mg/day over 10 days). Mean urinary aprotinin concentration ranged between 642 ± 135 (day 2) and 127 ± 16 (day 8) µg/mL . Aprotinin caused impaired sodium preservation under a low-salt diet while stimulating excessive hyperaldosteronism and unexpectedly, proteolytic activation of ENaC. Aprotinin inhibited proximal tubular function leading to glucosuria and proteinuria. Plasma urea and cystatin C concentration increased significantly under aprotinin treatment. Kidney tissues from aprotinin-treated mice showed accumulation of intracellular aprotinin and expression of the kidney injury molecule 1 (KIM-1). In electron microscopy, electron-dense deposits were observed. There was no evidence for kidney injury in mice treated with a lower aprotinin dose (0.5 mg/day). In conclusion, high doses of aprotinin exert nephrotoxic effects by accumulation in the tubular system of healthy mice, leading to inhibition of proximal tubular function and counterregulatory stimulation of ENaC-mediated sodium transport.

## Introduction

Aprotinin is a broad-spectrum serine protease inhibitor that was first isolated from cow parotis gland and pancreas in the 1930s. It is an unselective inhibitor of trypsin-like serine proteases such as trypsin, plasmin, plasma and tissue kallikreins, coagulation factor XII, and others [1]. Aprotinin was used as an antifibrinolytic agent in cardiac surgery to reduce blood loss associated with the extracorporal circuit [2]. Due to its peptide character, aprotinin is freely filtered and almost completely excreted by the kidneys. Between 2006 and 2008, studies appeared that found an increased risk for renal dysfunction and even mortality in patients undergoing cardiac surgery who were treated with aprotinin [3–6]. Renal dysfunction manifested as acute kidney injury ranging from small increases in serum creatinine concentration to dialysis-dependent renal failure. After early termination of the BART trial (Blood Conservation Using Antifibrinolytics in a Randomized Trial) due to increased mortality of aprotinin-treated patients [7], aprotinin was withdrawn from the market in 2007. Since then, the mechanisms leading to renal dysfunction remain ill-defined [8]. However, in the last years aprotinin is being reintroduced in Europe and Canada after reevaluation of the safety data, highlighting several methodological limitations [9].

Serine proteases are involved in the intrarenal regulation of the epithelial sodium channel ENaC that is expressed in the aldosterone-sensitive distal nephron and determines the final urinary sodium concentration [10, 11]. ENaC is a heterotrimer composed of three subunits named α, β, and γ, each participating in the formation of the channel pore [12]. A special feature of ENaC is its regulation by serine proteases, which remove inhibitory tracts from the α- and γ-subunits to fully activate the channel [10, 13, 14]. During maturation intracellular furin cleaves α-ENaC twice leading to the release of one inhibitory tract and γ-ENaC once. Final proteolysis and release of a second inhibitory tract from the γ-subunit requires an additional serine protease. Several renally expressed serine proteases such as membrane-anchored prostasin [15, 16] or tissue kallikreins [17] have been shown to be involved in proteolytic ENaC activation. An important fact is that they are all aprotinin-sensitive. Pathophysiologically, proteolytic activation of ENaC by aberrantly filtered serine proteases or proteasuria has been implicated in the sodium retention seen in nephrotic syndrome [18]. Our group has shown that treatment of nephrotic mice with the serine protease inhibitor aprotinin prevented ENaC-mediated sodium retention and edema formation as did the ENaC blocker amiloride [19–21].

Currently, the translation of the beneficial effects of aprotinin to patients with nephrotic syndrome is difficult due to the withdrawal of aprotinin and its association with renal dysfunction. We hypothesized that the mechanism for the negative renal effects of aprotinin might be related to the inhibition of serine proteases that are physiologically involved in ENaC regulation, thereby leading to kidney injury by sodium and volume loss. We therefore undertook this study to investigate the impact of aprotinin treatment on sodium handling in healthy wild-type mice under control and low-salt conditions.

## Materials and methods

### Mouse studies

Experiments were performed on 6-month-old wild-type 129S1/SvImJ mice of both sexes. Mice were kept up to five in Eurostandard type II long cages (365 mm × 207 mm × 140 mm, area 530 cm^2^) on a bedding from wood chips. Mouse houses made of plastic, wooden tunnels, and cellulose were also used as enrichment. The light–dark cycle was 12:12 h at an ambient temperature of 22–24 °C. Mice were fed a standard diet (ssniff, sodium content 0.24% corresponding to 104 µmol/g, Soest, Germany) with tap water *ad libitum*. To study urinary sodium excretion, all mice were housed singly in metabolic cages (diameter 20 cm, area 314 cm^2^, Tecniplast, Italy) for 2 days under a control diet (C1000, Altromin, Lage, Germany, sodium content 106 µmol/g). Thereafter, mice underwent implantation of custom-made pellets with a matrix-driven sustained release of aprotinin or placebo (Innovative Research of America, Florida, USA). These pellets were surgically implanted subcutaneously on the back of the mice under isoflurane anesthesia. After this operation, the wound conditions and the suture were checked several times. This route and mode of administration ensured that the kidneys were constantly exposed to aprotinin, which otherwise would rapidly be cleared by glomerular filtration. Subsequently, mice were studied in a 2 × 2 factorial design over 9 days in the metabolic cage (Supplementary Fig. 1a). Group 1 and 2 continued to receive the control diet with an aprotinin-containing or placebo pellet, respectively. Group 3 and 4 were switched to a low-salt diet (C1036, Altromin, Lage, Germany, sodium content 7 µmol/g) with or without aprotinin-containing or placebo pellet, respectively. The assignment to the respective group was random. The total aprotinin dose of the pellets was 5 or 20 mg corresponding to a daily dose of 0.5 or 2 mg bovine aprotinin (6000 kallikrein inhibitor U/mg, Loxo, Heidelberg, Germany) per day to be delivered over a maximum of 10 days. This dose was chosen from dose-finding studies in nephrotic mice [20]. At the end of the experiments, mice were anesthetized with isoflurane, exsanguinated, and euthanized by cervical dislocation. Afterwards kidneys were collected. In a subset of mice, ENaC-mediated transport reflected by the amiloride-sensitive natriuresis was determined from the urinary sodium excretion in 6 h after i.p. injection of vehicle (5 mL/kg [bw] injectable water) and amiloride (10 mg/kg bw) on the other day. No anesthesia was required for these injections. All animal experiments were conducted according to the ARRIVE 2.0 guidelines [22], the German law for the welfare of animals, and they were approved by local authorities (Regierungspräsidium Tübingen, approval number M 5/16).

### Laboratory measurements

Urinary protease activity from spot urine samples (treatment day 3) was measured using a universal peptide substrate library containing 19^5^ different pentapeptides (P-Check®, Panatecs, Germany). Each peptide contains a Förster resonance energy transfer pair consisting of the fluorophore 7-methoxycoumarinyl-4-acetyl and the quencher 2,4-dinitrophenol. Upon cleavage by any protease, a fluorescence signal can be detected at 405 nm emission after excitation at 320 nm. Proteolytic activity was measured from 5 µL urine samples incubated for 48 h at 37 °C with 10 µL substrate (P-Check®: 1 mg/mL) and 85 µL Tris-Buffered Saline in a total volume of 100 µL. Increase in fluorescence counts was detected on a fluorescence reader (Tecan, Spark®10 M). Values were expressed as relative fluorescence units [23].

Creatinine, glucose, phosphorus, and urea concentrations were measured with colorimetric assays (Labor + Technik, Berlin, Germany) and urinary sodium concentrations with flame photometry (Eppendorf EFUX 5057, Hamburg, Germany). Plasma sodium concentrations were measured using an IL GEM® Premier 3000 blood gas analyzer (Instrumentation Laboratory, Munich, Germany). ELISA kits were used to measure plasma aldosterone (IBL, Germany), plasma and urinary cystatin C (R&D systems, USA), and urinary aprotinin (Cloud corp, PRC). Urinary albumin was measured using a fluorometric kit against mouse albumin as standard (Active motif, Carlsbad, USA). Urinary amiloride concentration was measured fluorometrically according to Baer et al. [24].

### Western blot

Western blot analysis for γ-ENaC expression was performed from a membrane protein preparation of kidney cortex collected from placebo- or aprotinin-treated mice under a control or low-salt diet after 2 days. Half the kidney per mouse was sliced, and the cortex was dissected using a scalpel. Homogenization was performed using a Dounce homogenisator in 1 mL lysis buffer containing 250 mM sucrose, 10 mM triethanolamine HCl, 1.6 mM ethanolamine, and 0.5 mM EDTA at pH 7.4 (all Sigma) [21, 25]. During all preparation steps, aprotinin (40 µg/mL) and a protease inhibitor cocktail (final concentration 0.1 × stock; mini-complete, Roche) were present to avoid ENaC cleavage in vitro. Homogenates were centrifuged at 1000 ×*g* for removal of the nuclei. Subsequently, the supernatant was centrifuged at 20,000 ×*g* for 30 min at 4 °C, and the resulting pellet containing plasma membranes was resuspended and diluted to a concentration of 5 mg/L. This yielded higher ENaC signals compared to centrifugation at 300,000 ×*g*. Samples were deglycosylated using PNGaseF according to the manufacturer’s instructions (NEB, Ipswich, USA). First, samples were denaturated with a glycoprotein denaturing buffer. Samples were then incubated with glycobuffer, NP-40, and PNGaseF for 1 h at 37 °C. Subsequently, 20 µg of sample was loaded on a 7.5%-polyacrylamide gel for electrophoresis. After SDS-PAGE under reducing conditions, proteins were blotted onto a nitrocellulose membrane (Amersham GE healthcare) and probed for γ-ENaC with a rabbit antibody against rat γ-ENaC (SPC-405, Stressmarq, Victoria, Canada). Signals were detected using fluorescent secondary antibody labeled with IRDye 680RD and a fluorescence scanner (Licor Odyssey, Lincoln, USA). For loading control, total protein was measured using Revert Total Protein Stain (Licor, Lincoln, USA).

### Histological analyses from kidney tissue

Mouse kidneys were fixed in 4% paraformaldehyde (PFA)/0.1 M sodium phosphate buffer (PB) pH 7.4, dehydrated, and embedded in paraffin. Kidneys were cut into 2 µm thick slices and stained with periodic acid Schiff’s reagent. Sections were evaluated in a blinded fashion for signs of glomerular and tubular damage using semiquantitative analysis by glomerular sclerosis index (GSI) [26] and tubular sclerosis index (TSI) [27], respectively. Stainings were quantified using a score ranging from 0 to 3: no staining (0)–weak staining (1)–moderate staining (2)–strong staining (3).

For immunohistochemistry, paraffin-embedded formalin-fixed sections (2 µm) were deparaffinized and rehydrated using standard protocols. Antigen retrieval was achieved by boiling the samples for 2.5 min in target retrieval solution (Agilent Technologies Germany GmbH & Co. KG, Waldbronn, Germany) at 110 °C using a pressure cooker. Kidney sections were blocked for 15 min with normal goat serum diluted 1:5 in 50 mM tris(hydroxymethyl)-aminomethane(Tris), pH 7.4, supplemented with 1% (w/v) skim milk (Bio-Rad Laboratories, Munich, Germany), followed by incubation with the primary antibodies rabbit anti-γ-ENaC (1:50; as described above), mouse anti-aprotinin (1:500; Abcam, Cambridge, UK), or goat anti-KIM-1 (1:1000; Bio-Techne, Wiesbaden, Germany) overnight at 4 °C. After washing in Tris buffer (50 mM Tris, pH 7.4, supplemented with 0.05% (v/v) Tween 20 (Sigma-Aldrich, Munich, Germany; 3 × 5 min) secondary antibodies (a biotinylated goat anti-rabbit IgG, a biotinylated horse anti-mouse IgG, and a rabbit anti-goat IgG, all from Vector Laboratories, Burlingame, CA USA; 1:500) were applied for 30 min at room temperature. Sections were further processed using the VectaStain ABC kit according to the manufacturer’s instructions and DABImmpact (both Vector Laboratories) as substrate. Finally, the sections were counterstained in hemalaun, dehydrated, and mounted for observation using an Olympus Bx60 upright microscope.

### Electron microscopy

For electron microscopy, a small piece of mouse renal cortex was immersion fixed in 4% PFA and 1% (v/v) glutaraldehyde in 0.1 M PB. After post-fixation (same fixative o/n at 4 °C), tissue blocks were washed in 0.1 M PB, treated with OsO_4_ (0.5% for 60 min), and stained with uranyl acetate (1% w/v in 70% v/v ethanol). After dehydration, tissue blocks were embedded in Araldite resin (Serva Electrophoresis GmbH, Heidelberg, Germany). Eighty nanometer ultrathin sections were cut on an UC6 ultramicrotome (Leica, Wetzlar, Germany), rinsed in lead citrate buffer before analysis using a Zeiss Sigma scanning electron microscope using a STEM detector (Zeiss, Oberkochen, Germany).

### Data and statistical analysis

Data are provided as means with SEM. Data were tested for normality with the Anderson–Darling test, D’Agostino and Pearson test, Shapiro–Wilk test, and Kolmogorov–Smirnov test. Unpaired data from two groups were tested for significance using the unpaired *t*-test (normally distributed data) or Mann–Whitney U test (non-normally distributed data). Accordingly, paired data from two groups were tested with the paired *t*-test (normally distributed data) or Wilcoxon test (non-normally distributed data). Post hoc tests were done only if *F* was significant. Significance testing of unpaired normally distributed data from more than two groups was performed using one-way ANOVA with post hoc Dunnett’s and Sidak’s multiple comparison tests. Unpaired non-normally distributed data from more than two groups were analyzed by the Kruskal–Wallis test with the post hoc Dunn’s multiple comparison test. Correspondingly, paired normally distributed data from more than two groups were tested with repeated measure one-way ANOVA or mixed effects analysis with the post hoc Dunnett’s multiple comparison test. In this case, the Geisser–Greenhouse correction was always performed. Paired non-normally distributed data were tested using the Friedmann test and the subsequent post hoc Dunn’s multiple comparison test. To determine the correlation between two variables, the Pearson’s correlation coefficient *r* and the determination coefficient *r*^2^ were calculated. The Grubb’s test (*α* = 0.01%) was carried out once to identify a probable outlier (Fig. 6b). A *P* value < 0.05 by two-tailed testing was considered statistically significant in all analyses. Power analysis was carried out (*α* = 0.05 and 1 − *β* = 0.80) to estimate the number of animals used in this study, which yielded a minimum sample size of four mice per group. The respective specified group size consists of individual values, there are no technical replicates. All statistical calculations were performed using GraphPad Prism version 8.0.2 (GraphPad Prism, RRID: SCR_002798).

## Results

### Effect of aprotinin on urinary protease activity and ENaC-mediated sodium transport

**Fig. 1:**
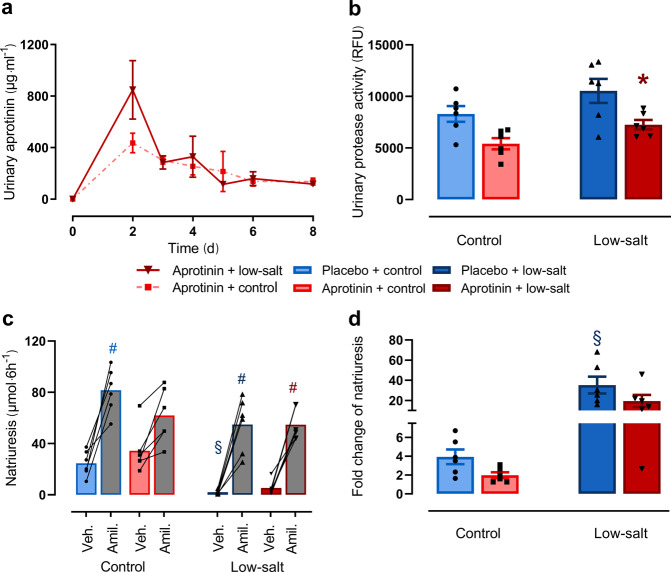
Effect of aprotinin on urinary protease activity and ENaC-mediated sodium transport. **a** Urinary aprotinin concentration after implantation of aprotinin-containing pellets with sustained release (2 mg/day over 10 days) in mice treated with control or low-salt diet. Note that there is a peak soon after implantation that can be explained by rapid release of unbound aprotinin. Data are only descriptive (each *n* = 2–4). **b** Urinary protease activity given in relative fluorescence units (RFU) in mice under control or low-salt diet with or without aprotinin treatment (each *n* = 6). **c**, **d** Natriuretic response to vehicle (Veh.) (5 mL/kg  bw) and amiloride (Amil.) (10 mg/kg  bw) under control or low-salt diet with or without aprotinin treatment (**c**). The calculated ratio corresponding to the slope in **c** indicating amiloride-sensitive natriuresis (**d**) (each *n* = 6). Data and statistical analysis: arithmetic means ± SEM. **b**, **d** One-way ANOVA followed by the Dunnett’s test; **c** One-way ANOVA followed by the Sidak’s test or Kruskal–Wallis test followed by the Dunn’s test. *P< 0.05, aprotinin vs. placebo treatment, ^§^*P* < 0.05, low-salt vs. control diet, ^#^*P* < 0.05 amiloride vs. vehicle treatment.

After implantation of aprotinin-containing pellets with sustained release (2 mg per day over 10 days), aprotinin was readily detected in the urine of wild-type mice (Fig. 1a). At day 2 after implantation, urinary concentration peaked and levelled off toward the end of the 10-day release duration. Aprotinin-treated mice excreted urine with reduced urinary protease activity under a sodium-replete control diet and low-salt diet (Fig. 1b). To investigate whether inhibition of urinary serine protease activity in aprotinin-treated mice interfered with the activity of the epithelial sodium channel ENaC, we determined the natriuretic response to the ENaC blocker amiloride and calculated the amiloride-sensitive sodium excretion as ratio of the values after vehicle and amiloride injection, which corresponds to the slope in Fig. 1c. Under a control diet, aprotinin-treated mice tended to have reduced amiloride-sensitive sodium excretion, which did not reach statistical significance (Fig. 1d). This tendency was not visible under a low-salt diet, and amiloride-sensitive sodium excretion was similarly increased in placebo- or aprotinin-treated mice. However, this finding was confounded by higher urinary amiloride concentration in aprotinin-treated mice (Supplementary Fig. S1b).

### Effect of aprotinin on sodium preservation and kidney function

Because ENaC activity determines the final urinary sodium concentration, it is essential for sodium homeostasis both under a sodium-replete and particularly under a low-sodium diet.

**Fig. 2:**
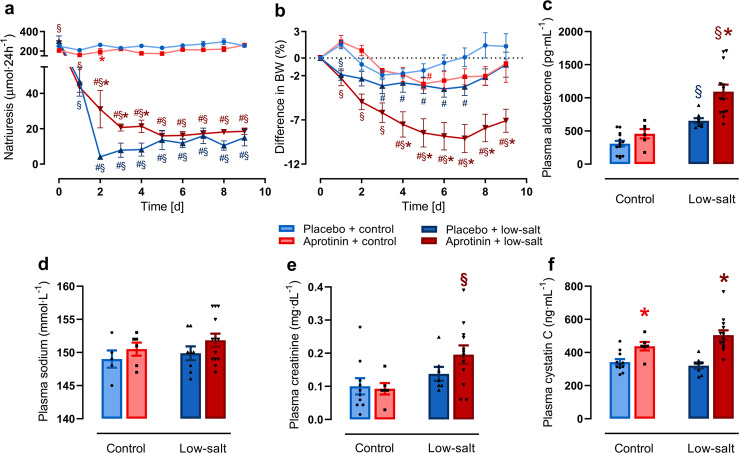
Effect of aprotinin on sodium preservation and kidney function. Course of 24-h urinary sodium excretion (**a**) and body weight (BW) (**b**) under control and low-salt diet in mice treated with placebo and aprotinin, respectively (placebo + control *n* = 8–14, aprotinin + control *n* = 6, placebo + low-salt *n* = 8, aprotinin + low-salt *n* = 13). Plasma aldosterone (**c**), sodium (**d**), creatinine (**e**), and cystatin C (**f**) concentration at the end of treatment (placebo + control *n* = 5–14, aprotinin + control *n* = 6, placebo + low-salt *n* = 8, aprotinin + low-salt *n* = 13). Data and statistical analysis: arithmetic means ± SEM. **a** Paired data: Wilcoxon test; mixed-effect analysis/Friedman test followed by Dunnett’s/Dunn’s test, unpaired data: unpaired *t*-test/Mann–Whitney test or One-way ANOVA/Kruskal–Wallis tests followed by Dunnett’s/Dunn’s test. **b** Paired data: Friedman test followed by the Dunn’s test, unpaired data: unpaired *t*-test/Mann–Whitney test or One-way ANOVA followed by the Dunnett’s test. **c**–**f** One-way ANOVA followed by the Dunnett’s test or Kruskal–Wallis test followed by the Dunn’s test. ^#^*P* < 0.05, significant difference to baseline, *P < 0.05, aprotinin vs. placebo treatment, ^§^*P* < 0.05, low-salt vs. control diet.

To investigate the effect of aprotinin on ENaC-mediated sodium conservation, we studied mice on a sodium-replete and low-sodium diet for 9 days in the metabolic cages. As shown in Fig. 2a, urinary sodium excretion was not affected by aprotinin over 9 days under a sodium-replete diet. Food and fluid intake as well as urine volume were similar in all groups (Supplementary Fig. S2a–c). Under a low-sodium diet, placebo-treated mice lowered urinary sodium excretion within 2 days and remained constant thereafter. In contrast, sodium conservation was impaired in aprotinin-treated mice during the first 5 days (Fig. 2a). Body weight dropped transiently in placebo- and aprotinin-treated mice under a control diet during the first days in the metabolic cages and stabilized thereafter (Fig. 2b). Body weight loss was pronounced under a low-sodium diet in placebo-treated mice and stabilized at the end of the study period. In contrast, aprotinin-treated mice under a low-sodium diet experienced enhanced bw loss that reached a new steady state at a lower level.

The plasma concentration of aldosterone, the master regulator of ENaC expression and activity, was not affected in aprotinin-treated mice under a control diet (Fig. 2c). Under a low-sodium diet, plasma concentration of aldosterone increased in placebo-treated mice whereas aprotinin-treated mice had excessively increased plasma aldosterone concentration, pointing to a compensatory stimulation. Plasma sodium concentration was constant through all groups (Fig. 2d).

Plasma creatinine, urea, and cystatin C concentration were measured as surrogates for glomerular filtration rate (Fig. 2e–f and Supplementary Fig. 2d). Under a control diet, plasma concentration of urea and cystatin C were significantly increased by aprotinin treatment while the plasma creatinine concentration was not altered. Under a low-sodium diet, all GFR surrogates were unaffected in placebo-treated mice; however, in aprotinin-treated mice all three surrogates were significantly increased, indicating kidney failure.

### Effect of aprotinin on renal expression of γ-ENaC

Upregulation of ENaC under a low-sodium diet is thought to involve increased membrane abundance and also proteolytic activation of the γ-subunit [28]. To investigate the effect of aprotinin on renal expression of γ-ENaC and its cleavage products, we analyzed kidneys from the same mice that were tested in metabolic cages on a sodium-replete and low-sodium diet using immunohistochemistry and Western blot.

**Fig. 3:**
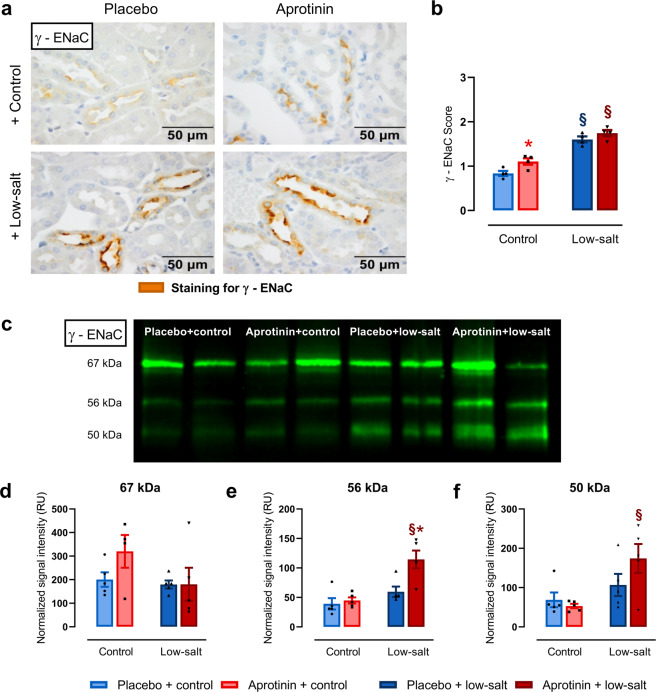
Effect of aprotinin on expression of γ-ENaC in kidneys from healthy mice. **a** Staining for γ-ENaC using immunohistochemistry in kidneys of mice treated with placebo and aprotinin under control and low-salt diet. Kidneys were harvested at day 9 (each *n* = 4). **b** Semiquantitative analysis of the obtained staining result (scale: no staining (0)–weak staining (1)–moderate staining (2)–strong staining (3); each *n* = 4). **c** Representative Western blot of lysates from kidneys of mice treated with placebo and aprotinin under control and low-salt diet. Kidneys were harvested at day 2. γ-ENaC was detected at 67, 56, and 50 kDa corresponding to full-length, furin-cleaved, and fully cleaved forms, respectively. **d**–**f** Densitometric analysis of the expression of the obtained bands (each *n* = 5). Signal intensity of the bands shown as relative units (RU) was normalized for total protein expression of the respective lane. Data and statistical analysis: arithmetic means ± SEM. **b** One-way ANOVA followed by the Dunnett’s test. **d**–**f** Unpaired *t*-test/Mann–Whitney test or One-way ANOVA/Kruskal–Wallis test followed by Dunnett’s/Dunn’s test. *P < 0.05, aprotinin vs. placebo treatment, ^§^*P* < 0.05, low-salt vs. control diet.

Under a control diet, tissue expression of γ-ENaC was barely detectable and was similarly upregulated under a low-sodium diet in placebo- and aprotinin-treated mice (Fig. 3a, b). Proteolytic activation of γ-ENaC was analyzed using Western blots from kidneys collected after 2 days to coincide with the largest difference in the urinary sodium excretion (Fig. 2a). As shown in Fig. 3c, γ-ENaC was detected at 67, 56, and 50 kDa corresponding to full-length, furin-cleaved, and fully cleaved forms, respectively. In placebo-treated mice under a control diet, the expression of fully cleaved γ-ENaC was low and tended to be increased under a low-sodium diet. Aprotinin treatment did not have an effect on the expression of fully cleaved γ-ENaC under control diet; however, upregulated its expression significantly under a low-sodium diet, indicating an unexpected stimulatory effect on proteolytic activation of γ-ENaC (Fig. 3f), which was in agreement with the increased amiloride-sensitive natriuresis shown in Fig. 1d.

### Effect of aprotinin on proximal tubular function

**Fig. 4:**
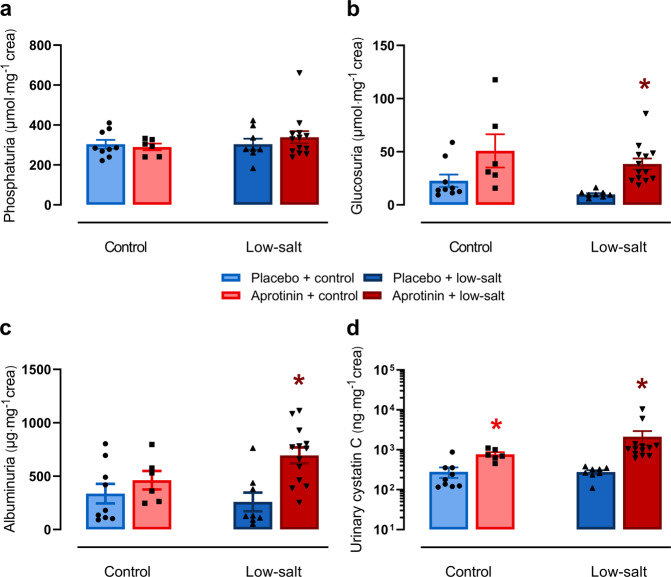
Effect of aprotinin on proximal tubular function. Urinary excretion of phosphate (**a**), glucose (**b**), albumin (**c**), and cystatin C (**d**) in mice treated with placebo and aprotinin after 8 days of a control or low-sodium diet (placebo + control *n* = 9, aprotinin + control *n* = 6, placebo + low-salt *n* = 8, aprotinin + low-salt *n* = 13). Data and statistical analysis: arithmetic means ± SEM. **a**–**d** Unpaired *t*-test/Mann–Whitney test or One-way ANOVA/Kruskal–Wallis test followed by Dunnett’s/Dunn’s test. *P < 0.05, aprotinin vs. placebo treatment.

To reconcile the finding of increased ENaC activity with impaired sodium preservation and bw loss under a low-sodium diet, we hypothesized that aprotinin treatment may impair sodium transport by causing dysfunction of the proximal tubule. Therefore, we investigated the effect of aprotinin on proximal tubular transport and measured urinary excretion of phosphate, glucose, albumin, and cystatin C in 24-h urine samples collected on day 4 and 8 (Fig. 4/Supplementary Fig. 3). Under control diet, aprotinin treatment had no significant effect on urinary excretion of phosphate whereas there was a tendency toward increased excretion of glucose and albumin (Fig. 4a–c). Urinary cystatin C excretion was significantly increased in aprotinin-treated mice under a control diet (Fig. 4d). Under a low-sodium diet, urinary excretion of these parameters was not affected in placebo-treated mice; however, aprotinin-treated mice exhibited significantly increased urinary excretion of glucose, albumin, and cystatin C. These effects were already evident in urine samples collected after 4 days of treatment (Supplementary Fig. 3).

### Effect of aprotinin on kidney histology

**Fig. 5:**
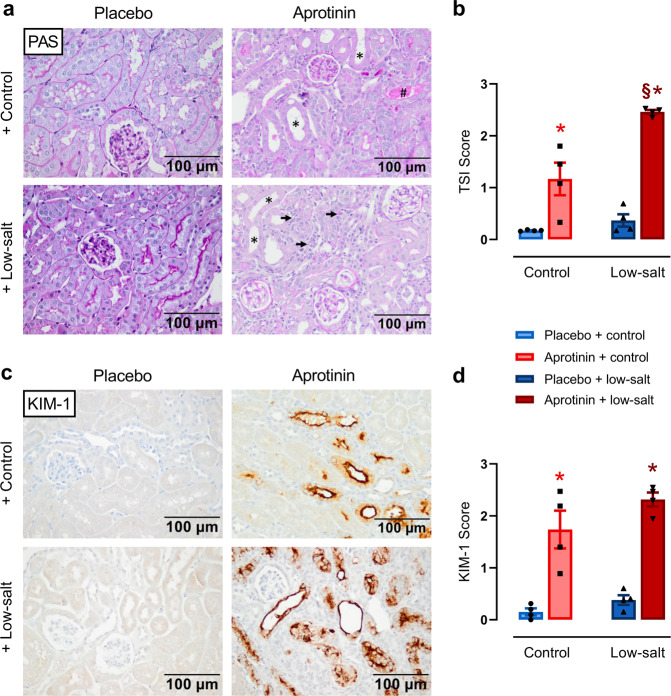
Effect of aprotinin on kidney histology. **a** PAS stainings from mice treated with placebo or aprotinin under a control or low-salt diet. As expected, kidneys from placebo-treated mice did not show any pathological changes. Kidneys from mice treated with 2 mg aprotinin per day showed moderate tubulointerstitial injury indicated by tubular dilatation and atrophy (marked by *) and rare tubular cast formation (marked by #). In contrast, aprotinin-treated mice on a low-sodium diet developed significantly more severe tubulointerstitial damage, mainly due to a marked increase in interstitial cells, presumably inflammatory cells and fibroblasts (marked by arrows). **b** Semiquantitative analysis of the observed changes using the tubular sclerosis index (TSI). **c** Immunohistochemical analysis of the expression of KIM-1, which is a marker of damage to the proximal tubule. **d** Semiquantitative analysis of KIM-1 expression. (**a**–**d** each *n* = 4). Data and statistical analysis: arithmetic means ± SEM. **b**, **d** One-way ANOVA followed by the Dunnett’s test. *P < 0.05, aprotinin vs. placebo treatment. ^§^*P* < 0.05, low-salt vs. control diet.

Under a control diet, kidneys from aprotinin-treated mice showed moderate tubulointerstitial injury indicated by tubular dilatation and atrophy and rare tubular cast formation (Fig. 5a). Under a low-sodium diet, aprotinin-treated mice developed significantly more severe tubulointerstitial damage, mainly due to a marked increase in interstitial cells, presumably inflammatory cells and fibroblasts. The TSI was increased in aprotinin-treated mice under both diets reaching higher values under a low-salt diet (Fig. 5b). The effect of aprotinin on glomerular integrity as analyzed by the GSI was minimal compared to placebo-treated mice, irrespective of the diet (0.61 ± 0.08 vs. 0.15 ± 0.03, *P* < 0.05). To substantiate the tubular damage more specifically, we stained for the expression of the kidney injury molecule 1 (KIM-1), which is a robust indicator of damage to the proximal tubule. As shown in Fig. 5c, d, KIM-1 staining was increased in aprotinin-treated mice under both diets.

### Accumulation of aprotinin in the kidney

**Fig. 6:**
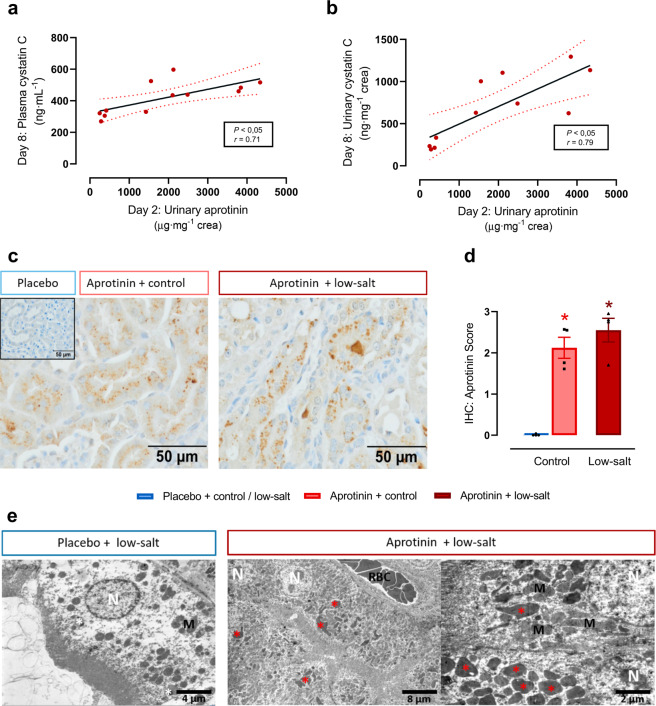
Accumulation of aprotinin in renal tissue. Correlation of urinary aprotinin concentration with plasma (**a**) and urinary cystatin C (**b**) concentration (each *n* = 11–12). **c** Immunohistochemical analysis of the tubular deposition and accumulation of aprotinin (each *n* = 4). **d** Semiquantitative analysis of aprotinin deposition (each *n* = 4). **e** Electron microscopy in different magnifications of proximal tubular cells from mice treated with placebo or aprotinin under a low-salt diet. In mice treated with placebo lysosomes in renal tubular cells appear normal in size and number (white asterisks). Whereas in mice treated with low-salt and aprotinin lysosomes were markedly increased in size and filled with electron-dense material (red asterisks). N nucleus, M mitochondria, RBC red blood cells (each *n* = 4). Data and statistical analysis: arithmetic means ± SEM. **a**, **b** Calculation of Pearson’s correlation coefficient *r*, *P* < 0.05; **b** one outlier removed (Grubb’s test–*α* = 0.01%). **d** Kruskal–Wallis test followed by the Dunn’s test. *P < 0.05, aprotinin vs. placebo treatment.

As shown in Fig. 6a, b, there was a significant correlation of urinary aprotinin concentration measured on day 2 with urinary and plasma cystatin C concentration measured on day 8. This could be related to accumulation of aprotinin in the proximal tubule, which is involved in the uptake and degradation of filtered peptides. To further investigate aprotinin accumulation in renal tissue, immunohistochemistry and electron microscopy were performed. As shown in Fig. 6c, d, aprotinin was readily detectable in proximal tubular cells under both control and low-salt diet consistent with an accumulation. Ultrastructural analysis of proximal tubular cells by electron microscopy revealed the presence of electron-dense material in the lysosomes that also were increased in size and number (Fig. 6e).

### Persistence and dose dependency of the aprotinin effect

**Fig. 7:**
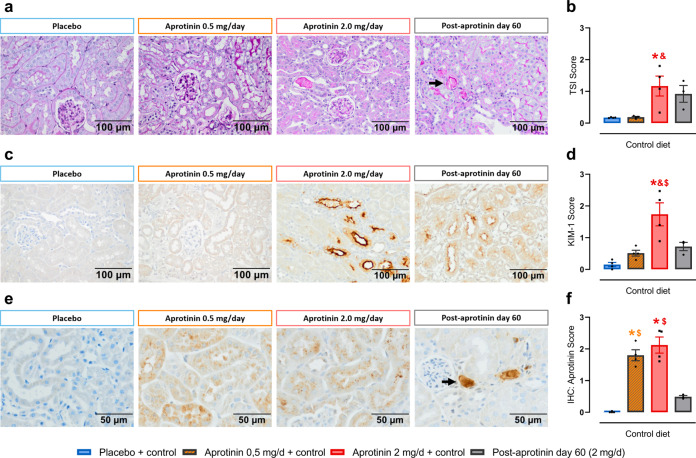
Persistence and dose dependency of the aprotinin effect. Sixty days post-aprotinin treatment (aprotinin 2 mg/day over 10 days) kidneys showed only minor signs of injury in the tubulointerstitial compartment as observed in PAS and KIM-1-stained renal sections (**a**–**d**). However, we sporadically observed severe focal tubular damage with PAS-positive luminal casts (**a**, arrow) that stained also positive for aprotinin (**e**, arrow) (**a**–**f** each *n* = 4). PAS staining (**a**) and tubular sclerosis index (TSI) (**b**). Immunohistochemistry for KIM-1 expression (**c**) and semiquantitative analysis (**d**). Immunohistochemistry for aprotinin content (**e**) and semiquantitative analysis (**f**). Data and statistical analysis: arithmetic means ± SEM. **b**, **d** One-way ANOVA followed by the Sidak’s test. **f** One-way ANOVA/Kruskal–Wallis test followed by the Sidak’s test/Dunn’s test. *P < 0.05, aprotinin vs. placebo treatment, ^&^*P* < 0.05, aprotinin 2.0 mg/day vs. 0.5 mg/day treatment, ^$^*P* < 0.05, aprotinin 2.0 mg/day vs. 60 days post-aprotinin treatment.

To investigate the persistence and dose dependency of the effects seen with 2 mg aprotinin per day, we performed additional analyses. First, we analyzed kidneys 60 days after end of aprotinin treatment. As shown in Fig. 7, these showed only minor signs of injury in the tubulointerstitial compartment as observed in PAS and KIM-1-stained renal sections (Fig. 7a, c). TSI and KIM-1 expression (Fig. 7b, d) were reduced (0.92 ± 0.26 and 0.72 ± 0.13, respectively); however, they were still higher than in untreated control kidneys (0.17 ± 0.01 and 0.15 ± 0.07, respectively). Still, there were severe focal tubular damage with PAS-positive luminal casts that stained also positive for aprotinin (Fig. 7a, e).

Second, we treated mice with aprotinin pellets releasing 0.5 mg/day (Supplementary Fig. 4a). As expected, this was followed by lower urinary aprotinin concentrations (Supplementary Fig. 4b). In contrast to the high dose, low dose aprotinin was not accompanied by increases in plasma cystatin C concentrations and urinary cystatin C excretion (Supplementary Fig. 4c, d). In renal tissue, there was no evidence for kidney injury as TSI and KIM-1 staining were not increased although aprotinin was detectable in proximal tubular cells (Fig. 7e, f).

## Discussion

Our study demonstrates that aprotinin treatment in high doses led to proximal tubular dysfunction in healthy wild-type mice as reflected by impaired sodium preservation, glucosuria, and proteinuria. Moreover, high-dose aprotinin treatment led to kidney injury and reduced glomerular filtration rate as evidenced by increased plasma creatinine, urea and cystatin C concentration, increased TSI, and most strikingly positive staining for KIM-1. It is likely that all these changes were caused by excessive accumulation and deposition of aprotinin in the tubular system observed in renal tissue. It is noteworthy that plasma and urinary cystatin C concentrations were positively correlated with the urinary aprotinin concentration suggesting a concentration-dependent effect. Aprotinin is a low molecular weight protein with a mass of 6512 Da, that is freely filtrated at the glomerulus and then taken up along with other low molecular proteins and albumin in the proximal tubule by receptor-mediated endocytosis involving the megalin/cubilin complex. Subsequently, these vesicles are fused with lysosomes where these proteins are degraded by proteases in an acidic environment. In addition, lysosomes are important determinants of cellular homeostasis by the mechanism of autophagy. Electron microscopy revealed that aprotinin accumulated intracellularly in the lysosomes of proximal tubular cells. It is well conceivable that this led to inhibition of protease activity and autophagy, which in turn induced injury of the proximal tubule. This injury pattern of aprotinin is reminiscent of Dent’s disease, which is caused by a hereditary defect in lysosomal function due to mutations of the renal vesicular Cl^−^/H^+^ antiporter CLC-5 [29].

The results with regard to sodium preservation were somewhat unexpected as the initial aim of the work was to demonstrate an inhibitory effect of aprotinin on proteolytic ENaC activation. We found that the opposite was true, indicating that aprotinin treatment did not inhibit but indirectly stimulated proteolytic ENaC activation as a counterregulatory response of the distal tubule to the decreased sodium reabsorption by the proximal tubule. This response was driven by markedly increased plasma aldosterone secretion in aprotinin-treated mice, which is expected to stimulate proteolytic ENaC activation [28]. One must assume that aprotinin treatment in healthy mice failed to directly inhibit proteolytic ENaC activation either by the fact that this happened intracellularly or that aprotinin did not reach sufficient local concentration at the cell surface to inhibit membrane-bound serine proteases. Among them, prostasin or channel activating protease 1 is a prominent glycosylphosphatidylinositol-anchored serine protease, which is aprotinin-sensitive and has been shown to be involved in proteolytic ENaC activation [15, 30]. Altogether, the effect of aprotinin on sodium transport in vivo was unanticipated and by far more complex as anticipated from its sole effect on proteolytic ENaC activation derived from in vitro ENaC expression systems [31].

The effect of aprotinin in healthy mice must be distinguished from that in mice with experimental nephrotic syndrome where aprotinin prevented ENaC-mediated sodium retention, most likely through inhibition of proteolytic ENaC activation by aberrantly filtrated serine proteases from the plasma or proteasuria [18, 20, 21]. The presence of large proteinuria is expected to compete with proximal tubular reabsorption of aprotinin, leading to increased intratubular and eventually urinary concentration of aprotinin. Indeed, the dose to achieve equivalent urinary aprotinin concentration was 1 mg per day in nephrotic mice of the same background compared to 2 mg in the present study [20]. Proteinuria is also expected to reduce accumulation in proximal tubular cells, thereby preventing proximal tubular damage, which is however, difficult to dissect from the effects of nephrotic syndrome. In nephrotic mice, aprotinin escaping reabsorption could better bind to aberrantly filtered proteases in the tubular lumen or reach the distal tubule to effectively prevent proteolytic ENaC activation. Therefore, it is unlikely that the prevention of sodium retention by aprotinin in nephrotic syndrome can be explained with inhibition of proximal tubular sodium reabsorption.

The results of the present study might be well applied to the human situation as the renal handling of aprotinin is almost identical. To compensate for rapid renal clearance, aprotinin is administered by a continuous infusion in humans, which in turn leads to a constant delivery to the tubule [32]. In the present study, we achieved continuous aprotinin exposure by using sustained release pellets. The results of the present study may provide an explanation why aprotinin was associated with renal adverse effects in patients, promoting its withdrawal from the market. The results indicate that aprotinin has a clear nephrotoxic potential in healthy mice, which was apparent with the higher dose. It is also remarkable that tubular deposition of aprotinin was still detectable 60 days after stopping treatment, indicating a very slow tissue clearance. In mice treated with the fourfold lower dose, we could not detect kidney impairment and injury, although aprotinin also accumulated in tubular cells. However, the treatment duration in mice was much longer than in patients who receive aprotinin for 24 h around cardiac surgery. As aprotinin is being reintroduced in clinical medicine in Europe and Canada, physicians should be aware of the nephrotoxic potential of aprotinin and try to minimize the risk, e.g., by choosing the lowest dose possible or stratifying the risk according to preexistent kidney disease.

The strength of this study lies in the fact that aprotinin treatment was done in conscious animals and achieved continuous aprotinin delivery to the kidney. This allowed studying its effect on tubular function and integrity. Previous studies had already demonstrated widespread tubular deposition of aprotinin in the proximal tubule and also collecting duct [33], which was confirmed in the present study. However, the functional consequences of aprotinin treatment have been only investigated in anesthetized animals so far, which is heavily confounded by narcosis and limited by the short treatment duration [34, 35]. The effects observed in the present study such as proximal tubular dysfunction, compensatory ENaC activation, and histological results were not investigated and not reported.

In conclusion, we demonstrate that high doses of aprotinin exert nephrotoxic effects in healthy mice, most likely caused by accumulation in tubular cells. This in turn leads to inhibition of proximal tubular function and counterregulatory stimulation of ENaC-mediated sodium transport. The results of the present study may provide an explanation why aprotinin was associated with renal adverse effects in patients.

## Supplementary information

Supplemental Figure 1-4
