# Supplementary material for: Renal effects of the serine protease inhibitor aprotinin in healthy conscious mice
Source: Acta Pharmacol Sin. 2021 Mar 23;43(1):111–20. doi: 10.1038/s41401-021-00628-1 (PMC8724274; doi:10.1038/s41401-021-00628-1)
Supplement: Supplementary file 1 — Supplemental Figure 1-4 [file 41401_2021_628_MOESM1_ESM.docx]

**Supplemental Figure 1. Experimental design and urinary amiloride excretion**

**a** Experimental design after subcutaneous implantation of placebo- or aprotinin containing (2 mg per day) pellets. Mice were treated with control or low-salt diet over 9 days in metabolic cages.

**b** Urinary amiloride concentration (each *n* = 6).

Data and statistical analysis: arithmetic means ± SEM.

* p < 0.05, aprotinin vs. placebo treatment.





**Supplemental Figure 2. Food, fluid intake and urine volume as well as plasma urea concentration during metabolic cage experiments with control and low**-**sodium diet.**

Food intake (**a**), fluid intake (**b**), urine volume (**c**) and plasma urea (**d**) concentration during the experiments (placebo + control *n* = 8-14, aprotinin + control *n* = 6, placebo + low-salt *n* = 8, aprotinin + low-salt *n* = 13).

Data and statistical analysis: arithmetic means ± SEM.

**#** p < 0.05, significant difference to baseline, ***** p < 0.05, aprotinin vs. placebo treatment, **§** p < 0.05, low-salt vs. control diet.





**Supplemental Figure 3. Effect of aprotinin on proximal tubular function after 4 and 8 days of treatment**

**a**-**d** Urinary excretion of phosphate (**a**), glucose (**b**), albumin (**c**) and cystatin C (**d**) in mice treated with placebo and aprotinin under 4 and 8 days of a control or low-sodium diet (day 4: each *n* = 4-5; day 8: placebo + control *n* = 9, aprotinin + control *n* = 6, placebo + low-salt *n* = 8, aprotinin + low-salt *n* = 13).

Arithmetic means ± SEM.

***** p < 0.05, aprotinin vs. placebo treatment at day 4 and day 8, respectively.





**Supplemental Figure 4. Dose dependency of the aprotinin effects**

**a** Experimental design.

**b** Urinary aprotinin concentration after implantation of sustained-release pellets with 0.5 mg or 2 mg aprotinin release per day. Data are only descriptive (each *n* = 2-4).

**c**, **d**: Plasma concentration (**c**) and urinary excretion of cystatin C (**d**) in mice treated with placebo, low dose (0.5 mg per day) and high dose (2.0 mg per day) aprotinin at the end of treatment (placebo + control *n* = 11, aprotinin 0.5 mg/d *n* = 7, aprotinin 2.0 mg/d *n* = 6).

Arithmetic means ± SEM.

***** p < 0.05, between aprotinin vs. placebo treatment, & p < 0.05, aprotinin 2 mg/d vs. aprotinin 0.5 mg/d treatment.
